# Multilevel analysis of undergoing clinical breast examination and its associated factors among mothers of reproductive age in Kenya: Kenyan Demographic and Health Survey 2022

**DOI:** 10.1371/journal.pone.0319183

**Published:** 2025-03-06

**Authors:** Mohammed Seid Ali

**Affiliations:** Department of Pediatrics and Child Health Nursing, School of Nursing, College of Medicine and Health Sciences, University of Gondar, Gondar City, Ethiopia; University of Oulu: Oulun Yliopisto, FINLAND

## Abstract

**Introduction:**

Breast cancer is one of the most common public health concerns among women around the world. The incidence of breast cancer is increasing in all areas of the world. It is the first cause of death from malignant tumors. Breast cancer in Sub-Saharan African countries is the number one cancer and the leading cause of cancer mortality among women. In low-income countries like Kenya, early screening programs, including clinical breast examination by health professionals, can identify women’s health status and risk of breast cancer. Therefore, this study was conducted to assess the uptake of clinical breast examination for cancer and to determine the associated factors among mothers of reproductive age.

**Methods:**

A total weighted sample of 10,267 mothers of reproductive age was included in this study. The data were taken from the recent Kenyan Demographic and Health Survey 2022. A multilevel multivariable logistic regression model was used to identify the associated factors associated with the uptake of clinical breast examination. In the multivariable multilevel analysis, the adjusted odds ratio (AOR) with a 95% CI was used to declare statistically significant associations with uptake among mothers of reproductive age in Kenya.

**Results:**

In Kenya, the overall prevalence of clinical breast examination uptake among mothers of reproductive age was 11.39%. In multilevel analysis, the significant factors associated with the uptake of clinical breast examination were the age of the mothers; age was significantly associated with the uptake of clinical breast examination; when compared with mothers aged 15–24 years, examination increased in those aged 25–34 years (AOR =  1.45; 95% CI (1.15–1.83)) and 34–49 years (AOR =  2.4; 95% CI (1.88–3.29)), when compared to no education, odds of examination increased in those with primary education (AOR =  2.0; 95% CI (1.19–3.37)) and secondary and higher (AOR =  2.67; 95% CI (1.56–4.57)), when compared to mothers who are unemployed, the odds of examination were higher among those who are employed (AOR =  1.42; 95% CI (1.16–1.74)), place of delivery; when compared to mothers who delivered at home, the odds of examination were higher among those who delivered at a health institution (AOR =  1.5; 95% CI (1.0–2.19)), when compared to those who are not exposed to television, odds of examination increased in those who were exposed to this form of media (AOR =  1.34; 95% CI (1.0–1.72)), when compared to those who travel on foot, odds of examination increased in those who used vehicles for transportation (AOR =  1.34; 95% CI (1.12–1.62)), and when compared to communities with a high level of literacy, the odds of examination increased in communities with a low level of literacy (AOR =  1.7; 95% CI (1.14–2.54)).

**Conclusion:**

In Kenya, the uptake of clinical breast examinations among mothers of reproductive age remains low. To address this, policymakers and stakeholders need to prioritize breast cancer screening programs to reduce mortality rates. The factors identified in this study are crucial for developing strategies to enhance clinical breast examination services, facilitating early detection and treatment of breast cancer.

## Introduction

Breast cancer is one of the most common public health concerns among women around the world. The incidence of breast cancer is increasing in all areas of the world. Female breast cancer has surpassed lung cancer as the most commonly diagnosed cancer, with an estimated 2.3 million new cases each year globally [1,2]. It is the most commonly diagnosed malignant tumor among women. It is the first cause of death from malignant tumors; an estimated 685,000 women died from breast cancer in 2020, one in every six cancer deaths in women [3,4]. Breast cancer is prevalent in both developed and developing countries, where it contributes to disproportionately high morbidity and mortality among women, particularly in low-income settings like sub-Saharan African (SSA) regions [5].

Breast cancer in SSA countries is the number one cancer and the leading cause of cancer mortality among women. In SSA, the incidence of breast cancer is expected to double by 2040 due to the expansion and aging of the population [6]. Breast cancer survival rates in Africa are poor and the five-year survival rate of breast cancer is nearly 50%, which means that in every two women diagnosed with breast cancer, one will die within five years after the diagnosis. However, in high-income countries, survival rates are increasing due to early diagnosis, appropriate care, and treatment. In developing countries like Kenya, approximately 80% of women are diagnosed in health facilities at the late stages of breast cancer, a locally advanced and metastatic disease [7,8].

Although less common, males can also develop breast cancer [9]. Approximately 99% of breast cancers occur in women, and 0.5–1% of breast cancers occur in men. While breast cancer predominantly affects females, it’s crucial to recognize that males can also be affected. Awareness, early detection, and tailored approaches are essential for both sexes [10]. This study investigated the female clinical breast examination because the demographic and health survey (DHS) data contains only maternal data.

The female breasts should be examined regularly to detect abnormalities early before ‘progression to breast cancer. The most common diagnostic modalities of breast cancer screening are breast self-examination, clinical breast examination by health care professionals, and mammography screening [11–13]. In high-income countries, mammography screening is most frequently used and considered to be the gold standard for early detection of breast cancer [14]. However, the accessibility of mammography is limited in low-income countries [15]; clinical breast examination by health care providers plays an important part in detecting breast cancer early to reduce morbidity and mortality in women [16,17]. Breast cancer encompasses various subtypes, each with distinct characteristics. The common types of breast cancer in Kenya are invasive ductal carcinoma, invasive lobular carcinoma, triple-negative breast cancer, luminal A and luminal B, and other subtypes [18].

In Kenya, health policy recommends clinical breast examination, as it offers an opportunity to provide education about breast health for women, though it is acknowledged that it is not a replacement for mammographic screening [19]. Clinical breast examination, performed by health professionals, has been a topic of debate regarding its effectiveness as a stand-alone screening modality for breast cancer. The Kenyan healthcare system has guidelines and programs in place for breast cancer screening. Kenya’s policies focus on health promotion, timely diagnosis, and comprehensive treatment. The National Cancer Screening Guidelines (2018) recommend population-based screening based on risk assessment and stratification. While clinical breast examination has limitations, it remains relevant, especially in resource-constrained settings [13]. Collaboration with mammography and tailored approaches can enhance breast cancer screening. It is important for the early detection of breast cancer [20]. Breast cancer screening by means of clinical breast examination by health professionals enables appropriate early intervention strategies such as early treatment and intensive counseling.

Breast health education and self-breast awareness are emphasized for early detection. For women with average breast cancer risk, the Kenyan National Screening Guidelines recommend regular screening based on risk assessment [21].

There are few studies about the uptake of clinical breast examination, and the screening rate among women of reproductive age in Africa [22,23],remains very low. In the last decade, incidents of breast cancer have been observed among both younger and postmenopausal women.

To the best of my knowledge, no national data exists on the uptake of clinical breast examinations among mothers of reproductive age in Kenya. The rate of clinical breast examinations for cancer screening remains low in the country. Therefore, this study aimed to assess the uptake of clinical breast examinations for cancer and identify the factors influencing it. The findings will help pinpoint actionable factors, guide future breast cancer screening efforts, and assist health policymakers in designing strategies to improve cancer control.

## Methods

### Study design and setting

This study was conducted in Kenya. Kenya is located in East Africa and borders Uganda to the west, Tanzania in the south, Sudan and Ethiopia in the north, and Somalia and the Indian Ocean in the east. A national cross-sectional study was conducted using Kenyan Demographic and Health Survey (KDHS) 2022 data. The Kenya National Bureau of Statistics (KNBS) and other stakeholders worked together to implement the 2022 KDHS. The data are from the seventh KDHS survey to be carried out. Data collection was conducted between February 17th and July 31st, 2022.

### Data source, extraction, sampling procedure, and study participants

The KDHS was the 7th to be carried out in Kenya, following similar surveys conducted in 1989, 1993, 1998, 2003, 2008–09, and 2014. The survey aimed to provide up-to-date information on socio-economic, demographic, nutrition, and health indicators to plan, monitor, and evaluate various health programs and policies. The sample for the 2022 KDHS was drawn from the Kenya Household Master Sample Frame (K-HMSF). This frame is used by the Kenya National Bureau of Statistics (KNBS) for household-based sample surveys in Kenya. After the 2019 Population and Housing Census, the K-HMSF was created, comprising a total of 129,067 enumeration areas (EAs). For the 2022 KDHS, a two-stage stratified sample design was employed: 1,692 clusters were selected in the first stage. These clusters were distributed across the country, with 1,026 clusters in rural areas and 666 in urban areas. Once the clusters were chosen, households within these clusters are selected randomly and a total of 42,300 households were chosen in the second stage. The clusters were developed through a process of household listing and georeferencing. Kenya is divided into 47 counties under the devolved system of government established by the Constitution of Kenya in 2010. Interviews were conducted only in the pre-selected households and clusters; no replacement of the pre-selected units was allowed during the survey data collection stages. Only mothers of reproductive age with biological children were included in our analyses.

### Study variables

The outcome variable in this study was clinical breast examination, which was determined by the KDHS question “breast examined for cancer by a health care provider.” The response was dichotomized as “yes” if the mothers had undergone breast examination by a health care provider and “no” if the mothers had undergone breast examination by a health care provider within the study period. The independent variables included in the study were maternal age, marital status, place of residence, educational status, religion, watching television, listening to radio, wealth status, occupational status, mode of transportation to health facilities, preceding birth interval, place of delivery, number of antenatal care (ANC) visits, visits by community health workers (CHWs), and service/information provided by CHWs.

### Data management and analysis

To restore the survey’s representativeness, the sample weights were applied to compensate for the unequal probability of selection between the strata. STATA software (version 17) was used to conduct descriptive statistics and logistic regression analysis. A binary logistic regression model was fitted. Multivariable logistic analysis was employed to identify the associated factors associated with clinical breast examination. Both bivariable and multivariable binary logistic regressions were conducted. Variables with a p-value <  0.2 in the bivariable analysis were eligible for multivariable analysis. In the multivariable analysis, an adjusted odds ratio (AOR) with a 95% confidence interval (CI) was reported, and variables with a p-value <  0.05 were considered as factors with a statistically significant association with clinical breast examination for cancer. For the assessment of cluster-level variability in clinical breast examination, random effect analysis was carried out because the data in DHS is hierarchal and clustered in nature; there are individual level variables and community-level variables. Variables were analyzed at the individual level, at the community level and at the overall level using four models: the null model, the individual-level model, the community-level model, and the multilevel model. The model with the best fit had the lowest deviance and the highest log likelihood ratio (LLR). We have clearly presented in the random effect analysis the intra-class correlation coefficient (ICC), variance, LLR proportion change in variance (PCV), median odds ratio (MOR), and deviance. To assess multicollinearity, the variance inflation factor (VIF) was used [24].

### Ethical approval and consent to participate

This study was based on secondary data analysis of publicly available national survey data from the DHS program. Ethics approval permission was obtained from MEASURE DHS program to use the data set for this study. We requested permission to download the DHS program, and it was granted. It uses data from http://www.dhsprogram.com. During DHS program, written or verbal consent were obtained before data collection from participants. The Institutional Review Board and the Research and Ethics Committee of the Kenyan Ministry of Health gave their approval to the survey protocol. All procedures were carried out in accordance with the Declaration of Helsinki on ethical principles for conducting human research, in addition to receiving ethics approval and informed consent.

## Results

### Socio-demographic-related characteristics of the participants

A total of 10,267 mothers of reproductive age participated in this study. Among the total, nearly half (49.98%) of the participants were in the category of 25–34 years old, and about one-third (33.36%) of the mothers lived in urban areas. Regarding maternal educational status, about 23.94% of the total participants did not attend formal education. The majority (75.66%) of the participants were married. Among the total participants, nearly half (49.27%) of the mothers were unemployed (Table 1).

**Table 1:** Socio-demographic related characteristics of the participants, KDHS 2022 (n =  10,267).

Variable	Response	Frequency	Percent (%)
Age of mother in years	15–24	2788	27.15
	25–34	5131	49.98
	35–49	2348	22.87
Place of residence	Urban	3425	33.36
	Rural	6842	66.64
Mother’s education	No education	2458	23.94
	Primary education	3544	34.52
	Secondary education	2871	27.96
	Higher	1394	13.58
Marital status	Unmarried	782	7.62
	Married	7768	75.66
	Ever married	1717	16.72
Mother’s employment status	Unemployed	5059	49.27
	Employed	5208	50.73
Religion	Catholic	1679	16.35
	Protestant	3082	30.02
	Evangelical	2045	19.92
	African churches	689	6.71
	Islam	2295	22.35
	Others^*^	477	4.65

* Others = Traditionalists, No religion, Hindu, Orthodox.

### Socio-economic and maternal-related variables of the participants

Among the total participants, nearly one-third (33.49%) of the respondents were from the poorest households in terms of their economic status. The majority (71.29%) of the mothers traveled to the health facility on foot. Nearly one-third (32.39%) of the mothers had a history of delivery to their homes. About 61.35% of the mothers had visited the health facility more than four times for their ANC follow-up. About 35.33% of the participants had aware clinical breast examinations for cancer. About 56.02% and 42.38% of the participants had media exposure through radio and television, respectively (Table 2).

**Table 2:** Socio-economic and maternal related variables of the participants, KDHS 2022 (n =  10,267).

Variable	Response	Frequency	Percent (%)
Wealth index	Poorest	3438	33.49
	Poorer	1744	16.99
	Middle	1743	16.98
	Richer	1895	18.46
	Richest	1447	14.09
Mode of transportation	Vehicle	2948	28.71
	On foot	7319	71.29
Place of delivery	Home	3325	32.39
	Health institutions	6942	67.61
Preceding birth interval	<24 months	2598	25.30
	≥24 months	7669	74.70
ANC visits	No visit	399	3.89
	<4 visits	3569	34.76
	≥4 visits	6299	61.35
Aware can examine breasts for cancer	No	6640	64.67
	Yes	3627	35.33
Visited by community health workers	No	9469	92.23
	Yes	798	7.77
Radio exposure	No	4515	43.98
	Yes	5752	56.02
Television exposure	No	5916	57.62
	Yes	4351	42.38

### The prevalence of undergoing clinical breast examinations by health care providers

**Fig 1:**
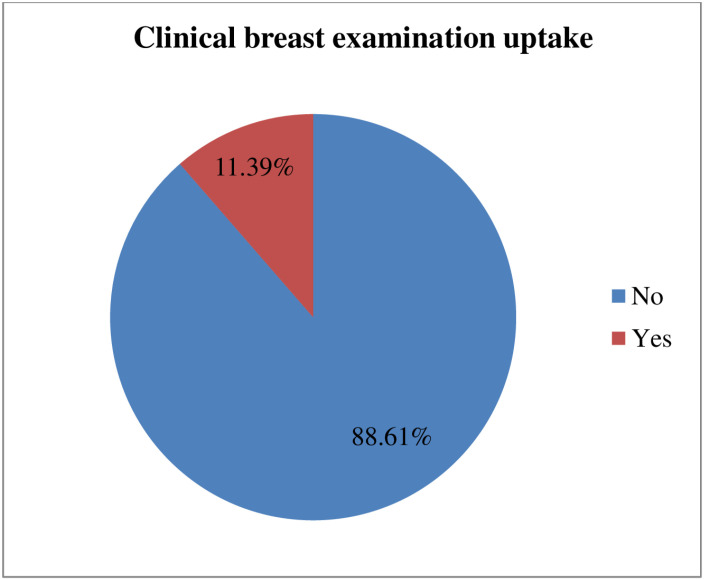
The prevalence of clinical breast examination uptake among women of reproductive age in Kenya (n= 10,267) KDHS 2022.

In this study, only one thousand one hundred sixty-nine (11.39%) of the mothers were undergone a clinical breast examination for cancer by a health care provider in the health institutions, with a 95% CI (10.78–12.02%) (Fig 1).

### Random effect analysis for cluster variability and model fitness

The total variation in the uptake of clinical breast examinations was attributable to clustering. The clustering effect and model estimation are shown in Table 3. The result of the null model showed that there was significant variability in the odds of uptake of clinical breast examination, with a community variance of 2.2. In addition, the MOR was 3.83; meaning that the odds of uptake of clinical breast examination might increase when respondents moved from low-to high-exposed communities. This indicated the existence of significant heterogeneity in the uptake of clinical breast examinations across different clusters. This variability is directed toward conducting multilevel analysis to identify the determinant factors associated with clinical breast examination. Model III was the best-fitted model, with the lowest deviance and the highest LLR. In model III (adjusted for both individual and community variables), the variance (0.34) remained significant (p-value < 0.001). The PCV in this model was 84.3%, which shows that 84.3% of the cluster variance observed in the null model was explained by both individual-level and community-level variables (Table 3).

**Table 3:** Model estimation for associated factors associated with clinical breast examination among women of reproductive age in Kenya (n = 10,267) KDHS 2022.

Parameter	Null model	Model I	Model II	Model III
Variance	2.206946	0.3391365	1.460894	0.3464902
ICC	40.15%	9.34%	30.75%	9.52%
MOR	3.83	1.50	3.12	1.52
PCV	Reference	84.6%	33.8%	84.3%
**Model fit**				
LLR	−3412.4527	−1778.4055	−3295.3848	−1774.3354
Deviance	6,824.9054	3,556.811	6,590.7696	3,548.6708

ICC, intra-cluster correlation; LLR, log-likelihood ratio; MOR, median odds ratio; PCV, proportional change in variance.

### Multilevel analysis of the factors associated with the uptake of clinical breast examination

The results of the multilevel logistic analysis are presented in Table 4 as the AOR and 95% CI. In model I, the associated factors were identified at the individual/household level. There was no multicollinearity between independent variables, with a mean VIF of 2.82 (the minimum and maximum VIF were 1.01 and 7.78, respectively). The significant factors associated with clinical breast examination at the individual level were the age of the mothers; 25–34 years (AOR =  1.46; 95 CI (1.16–1.85), 34–49 years (AOR =  2.55; 95% CI (1.93–3.37)), maternal education; primary education (AOR =  2.9; 95% CI (1.88–4.53)), secondary and higher (AOR =  3.96; 95% CI (2.52–6.25)), employment status (AOR =  1.47; 95% CI (1.21–1.79)), place of delivery (AOR =  1.56; 95% CI (1.06–2.29)), wealth index; richer (AOR =  1.4; 95% CI (1.1–1.88)), television exposure (AOR =  1.36; 95% CI (1.1–1.74)), and mode of transportation; vehicle (AOR =  1.35; 95% CI (1.1–1.63)).

**Table 4:** Multilevel analysis of the associated factors associated with clinical breast examination among women of reproductive age in Kenya (n = 10,267) KDHS 2022.

Variable	Response	Model I	Model II	Model III
Age of mother in years	15–24	Reference		Reference
	25–34	1.46 (1.16–1.85)	–	**1.45 (1.15–1.83)** ^*^
	35–49	2.55 (1.93–3.37)	–	**2.4 (1.88–3.29)** ^*^
Mother education	No education	Reference		Reference
	Primary education	2.9 (1.88–4.53)	**–**	**2.0 (1.19–3.37)** ^*^
	Secondary and higher	3.96 (2.52–6.25)	**–**	**2.67 (1.56–4.57)** ^*^
Mother’s employment	Employed	1.47 (1.21–1.79)	–	**1.42 (1.16–1.74)** ^*^
	Unemployed	Reference		Reference
Marital status	Unmarried	Reference		Reference
	Married	0.94 (0.68–1.31)	–	0.97 (0.7–1.34)
	Ever married	1.1 (0.79–1.64)	–	1.15 (0.8–1.67)
Place of delivery	Home	Reference		Reference
	Health institution	1.56 (1.06–2.29)	–	**1.5 (1.0–2.19)** ^*^
ANC visits	No visit	Reference		Reference
	<4 visits	0.86 (0.39–1.91)	–	0.85 (0.38–1.87)
	≥4 visits	1.1 (0.48–2.34)	–	1.01 (0.47–2.31)
Wealth index	Poorer	Reference		Reference
	Middle	1.02 (0.76–1.37)	–	0.98 (0.71–1.35)
	Richer	1.4 (1.1–1.88)	–	1.37 (0.94–1.99)
Radio exposure	No	Reference	–	Reference
	Yes	1.1 (0.87–1.33)	–	1.0 (0.84–1.29)
Television exposure	No	Reference		Reference
	Yes	1.36 (1.1–1.74)	–	**1.34 (1.0–1.72)** ^*^
Mode of transportation	Vehicle	1.35 (1.1–1.63)	–	**1.34 (1.12–1.62)** ^*^
	On foot	Reference	–	Reference
Visited by CHW	No	0.7 (0.56–1.09)	–	0.7 (0.55–1.07)
	Yes	Reference		Reference
Place of residence	Urban	**–**	1.23 (0.97–1.56)	0.95 (0.73–1.22)
	Rural	–	Reference	Reference
Community illiteracy level	High	–	Reference	Reference
	Low	–	5.4 (3.98–7.38)	**1.7 (1.14–2.54)** ^*^
Community poverty level	High	–	Reference	Reference
	Low	–	1.0 (1.7–2.19)	1.1 (0.81–1.44)

* Statistically significant at a p-value <  0.05.

At the community level, the significant factors associated with the uptake of clinical breast examination were low community illiteracy level (AOR =  5.4; 95% CI (3.98–7.38)) and low community poverty level (AOR =  1.0; 95% CI: (1.7–2.19)).

In multivariable multilevel analysis, the significant factors associated with the uptake of clinical breast examination were the age of the mothers; 25–34 years (AOR =  1.45; 95% CI (1.15–1.83)), 34–49 years (AOR =  2.4; 95% CI (1.88–3.29)), the age of the mothers older than 25 years were more likely to undergo clinical breast examination as compared to younger age, maternal educational status; primary education (AOR =  2.0; 95% CI (1.19–3.37)), secondary and higher (AOR =  2.67; 95% CI (1.56–4.57)), the odds of undergoing clinical breast examination was higher among educated mothers as compared to mothers who had no formal education, employment status (AOR =  1.42; 95% CI (1.16–1.74)); employed mothers had higher odds of undergoing clinical breast examination as compared to their counterparts, place of delivery (AOR =  1.5; 95% CI (1.0–2.19)), mothers who deliver in the health institution had higher odds of undergoing clinical breast examination as compared to home delivery, television exposure (AOR = 1.34; 95% CI (1.0–1.72)); those mothers who had media exposure through television had higher odds of undergoing clinical breast examination as compared to their counterparts, mode of transportation (AOR = 1.34; 95% CI (1.12–1.62)); those mothers who travelled to health facility by vehicle were 1.34 times more likely to undergo clinical breast examination as compared to those who travelled on foot, and community illiteracy level (AOR = 1.7; 95% CI 1.7(1.14–2.54)); low level of community illiteracy had higher odds to undergo clinical breast examination as compared to their counterparts (Table 4).

## Discussion

This study investigated the uptake of clinical breast examination and its associated factors among mothers of reproductive age in Kenya using recent data from KDHS 2022. The prevalence of clinical breast examination uptake among mothers of reproductive age in Kenya was 11.39% with a 95% CI (10.78–12.02). The prevalence of clinical breast examination uptake was very low among mothers. Clinical breast examination was associated with socio-demographic, socio-economic, and maternal factors. Studying the status of clinical breast examination uptake is an important indicator of maternal health care quality and the country’s healthcare system.

The prevalence of clinical breast examination uptake among mothers of reproductive age in Kenya was lower than in other studies conducted in Saudi Arabia 27.4% [25], Turkey 19.0% [26], Vietnam 51% [27], and the United Arab Emirates 55.2% [28]. This discrepancy might be due to socio-demographic and economic variation between Kenya and other countries. This might also be due to the variation in the health care system and cancer screening efforts like clinical breast examination. Clinical breast examination uptake varies based on context, awareness, and healthcare systems. Since, the uptake of clinical breast examination is affected by socio-demographic factors, socio-economic factors, and the accessibility of health care services [29–31].

The prevalence of clinical breast examination uptake among mothers of reproductive age in Kenya was slightly higher than other studies conducted in Tanzania 0.9% [32], Ghana 10.1% [33], and Lesotho 9.73% [34]. The higher prevalence of clinical breast examination uptake among mothers of reproductive age in Kenya compared to studies conducted in Tanzania, Ghana, and Lesotho could be attributed to various factors. These might include differences in healthcare infrastructure, awareness campaigns, cultural attitudes, and socioeconomic conditions. However, specific reasons would require further investigation and analysis [35].

The multilevel analysis identified the associated factors associated with the uptake of clinical breast examination. In this study, being older was positively associated with the uptake of clinical breast examination. Older women often visit healthcare facilities more frequently due to age-related conditions, which can lead to more routine screenings. These visits provide opportunities for early detection of various health issues, even if the primary reason for the visit is unrelated to the screening itself. This finding was supported by other studies conducted in Ireland, China, and Turkey [36–38]. The age discrepancy in clinical breast examination may be influenced by the information provided and the breast cancer screening programs, which tend to encourage older mothers more than younger ones. It is also due to the variation in health care system and economic status between Kenya and other countries. The risk of breast cancer does tend to increase with age, and regular clinical breast examinations play a crucial role in early detection. As mother’s age, their breast tissue changes, and the likelihood of developing breast cancer rises. This could also be due to the fact that the habit of clinical breast examination will be higher as compared to younger mothers. In many countries, breast cancer screening programs like mammography started for women aged 40 years and older [39,40].

The educational status of mothers affects their breast cancer screening attitude and behavior. This study revealed that mothers with higher educational status are more likely to undergo clinical breast examinations. Reproductive-age women who attended primary or secondary education had higher odds of undergoing clinical breast examination as compared to uneducated mothers. Based on this finding of the study, the education of mother’s has great importance for breast cancer screening programs. This finding was supported by other studies [41–43]. The possible explanation might be educational status of mothers significantly influences their attitude and behavior toward breast cancer screening. Mothers with higher education levels tend to be more aware of the importance of regular screenings, understand the risks, and are more likely to participate in screening programs. Conversely, lower educational attainment may lead to limited awareness, misconceptions, and barriers to accessing healthcare services. Promoting education and awareness can positively impact breast cancer prevention and early detection [44]. The finding that the odds of attending clinical breast examinations are higher among communities with lower literacy levels, while also noting that the odds of uptake increase with higher maternal education levels, presents an interesting paradox. One possible explanation is that in communities with lower literacy levels, there might be more targeted and accessible health campaigns or community-based interventions that encourage women to attend clinical breast examinations. These efforts might be more effective in reaching and educating women about the importance of breast cancer screening, even if their overall literacy levels are lower. On the other hand, higher maternal education levels are generally associated with better health literacy, which means these women are more likely to understand the importance of preventive health measures, including clinical breast examinations. This could explain why uptake increases with higher maternal education levels. In essence, while overall community literacy might be lower, targeted health interventions can still effectively promote clinical breast examinations attendance. Conversely, higher maternal education levels typically lead to better health outcomes due to increased health literacy and awareness.

The employment status of mothers was another factor associated with the uptake of clinical breast examinations. Those mothers who were employed were more likely to undergo clinical breast examinations as compared to unemployed mothers. This finding was supported by other studies [45,46]. The employment status of mothers can determine their socioeconomic status. Their socioeconomic status affects their health care accessibility, like transportation and covering health care costs. This might be due to the fact that mothers who are employed have better access to health care costs, transportation to the health facility, better media exposure, and access to health related information in the working area.

Regarding the type of place of delivery, health institution delivery was positively associated with the uptake of clinical breast examination. Those mothers who delivered in health institutions were more likely to undergo a clinical breast examination as compared to those who delivered at home. Clinical breast screening services will be provided to mothers mainly in health institutions. This finding was supported by another study [47]. This may be due to pregnant mothers delivering at health institutions receiving more, or better, information about breast screening programs, including clinical breast examination, as compared to mothers who delivered at home. This might also be due to the fact that in the health institution, clinical examination and breast screening services are usually provided by health care professionals to all pregnant mothers attending health institution. Therefore, institutional delivery helps reproductive-aged mothers in many aspects of their health status.

Access to transportation by vehicle was positively associated with the uptake of clinical breast examinations. This implies that distance to a health facility impacts the likelihood of mothers undergoing clinical breast examinations, as those who traveled on foot had lower odds of participating. This finding was consistent with other studies [48–50]. Mothers living in remote areas often face challenges accessing healthcare services due to the long distances they must travel to reach health facilities. While those who can travel by vehicle can arrive promptly, mothers who walk to the facility may find it difficult to cover such extended distances for healthcare. Transportation barriers, such as distance to treatment centers, lack of reliable public transportation, and physical mobility limitations, can hinder mothers from seeking healthcare services[51]. Therefore, the mode of transportation to the health facility affects the uptake of clinical breast examinations.

This finding indicates that media exposure through television encourages the likelihood of clinical breast cancer screening program attendance among reproductive aged mothers. This finding was in agreement with the other studies [52–54]. Mass media like television are very important to promote health-seeking behavior. This is due to the fact that mass media like television can create a better opportunity to disseminate health-related information to the general population at large. There is also a possible explanation for this situation: those mothers who are exposed to television might be able to get information related to health care services, like the need for breast cancer screening. Therefore, the uptake of clinical breast examinations is affected by the media exposure through television. The intersectionality of factors influencing the uptake of clinical breast examinations is crucial. Various studies highlight multiple factors that interplay, affecting clinical breast examinations among women. For instance, socioeconomic status, education level, awareness of breast cancer, and access to healthcare facilities are significant determinants [55,56].

### Strengths and limitations of the study

This study was conducted based on a large and nationally representative data set. The study employed advanced statistical analyses like random effects and fixed effects multilevel analyses. It is important to get more informative and intervention-based findings. However, the data of this study was limited to reproductive-age mothers (15–49 years), although the risk of breast cancer includes those older than 49 years. To include important maternal related variables, this study also used only mothers, with children contributing data to maternal outcomes. A further limitation is the absence of postmenopausal women and women with no biological children from the dataset, even though these groups are also important risk groups for breast cancer as both advanced age and nulliparity are associated with increased risk of breast cancer. Due to the cross-sectional nature of the study, some variables that were aggregated at the community level might potentially mask within-community variations. Future researchers can address the limitations to enhance mother’s health outcomes.

## Conclusion

The uptake of clinical breast examination among mothers of reproductive age in Kenya was low.

Factors significantly associated with the uptake of clinical breast examination were higher age, greater levels of education, employment, institutional delivery, media exposure (television), vehicle transportation, and low community illiteracy.

Given these findings, policymakers and stakeholders should prioritize breast cancer screening services, emphasizing education, awareness, and accessibility to improve early detection and treatment of breast cancer. It is recommended to place special emphasis on the above associated factors that are an important input to developing strategies for improving breast cancer screening services as well as early detection and treatment of breast cancer in the country.

## Supporting information

S1 File A supplementary file showing the data set for this study. (XLS)

## Abbreviations

ANC: antenatal care
AOR: adjusted odd ratio
CI: confidence interval
CHW: Community Health Workers
DHS: Demographic and Health Survey
KDHS: Kenyan Demographic and Health Survey
KNBS: Kenya National Bureau of Statistics
K-HMSF: Kenya Household Master Sample Frame
ICC: intra-cluster correlation
LLR: log-likelihood ratio
MOR: median odds ratio
PCV: proportion Chang in variance
SSA: sub-Saharan Africa
VIF: variance inflation factor.
